# α4 Nicotinic Acetylcholine Receptors in Lipopolysaccharide-Related Lung Inflammation

**DOI:** 10.3390/ijms252011305

**Published:** 2024-10-21

**Authors:** Jeffrey D. Ritzenthaler, Walter H. Watson, Jesse Roman

**Affiliations:** 1 Department of Medicine, Division of Pulmonary, Allergy and Critical Care Medicine and the Jane & Leonard Korman Respiratory Institute, Thomas Jefferson University, Philadelphia, PA 19107, USA; 2 Department of Medicine, Division of Gastroenterology, Hepatology & Nutrition, University of Louisville, Louisville, KY 40292, USA; bert.watson@louisville.edu; 3 Department of Pharmacology & Toxicology, University of Louisville, Louisville, KY 40292, USA

**Keywords:** redox stress, nicotinic receptors, lung injury, monocytic cells, sepsis

## Abstract

Sepsis remains an important healthcare challenge. The lungs are often affected in sepsis, resulting in acute lung injury characterized by inflammation. Mechanisms involving lipopolysaccharide (LPS) stimulation of toll-like receptor (TLR) signaling with induction of proinflammatory pathways have been implicated in this process. To date, however, studies targeting these pathways have failed to improve outcomes. We have found that LPS may also promote lung injury through the activation of α4 nicotinic acetylcholine receptors (α4 nAChRs) in immune cells. We observed increased expression of α4 nAChRs in human THP-1 monocytic cells exposed to LPS (100 ng/mL, 24 h). We also observed that LPS stimulated the expression of other relevant genes, including tumor necrosis factor-α, interleukin-1β, plasminogen activator inhibitor-1, the solute carrier family 7 member 11, extracellular superoxide dismutase, and transforming growth factor-β1. Of interest, dihydro-β-erythroidine hydrobromide (DHβE), a specific chemical inhibitor of α4 nAChRs, inhibited the LPS-induced expression of these genes. We generated mice with a global knockout mutation of the α4 nAChR subunit in the C57BL/6 background using CRISPR/Cas9 technology. The lungs of these LPS-treated animals demonstrated a reduction in the expression of the above-mentioned genes when compared with the lungs of wild-type animals. In support of the role of oxidative stress, we observed that LPS induced expression of the cystine transporter Slc7a11 in both THP-1 cells and in wild-type mouse lungs. The effects of LPS on THP-1 cells were blocked by the thiol antioxidant N-acetylcysteine and mimicked by redox stress. Importantly, the induction of IL-1β by redox stress was inhibited by the α4 nAChR inhibitor DHβE. Finally, we showed that LPS stimulated calcium influx in THP-1 cells, which was blocked by the α4 nAChR inhibitor. Our observations suggest that LPS promotes lung injury by stimulating redox stress, which activates α4 nAChR signaling and drives proinflammatory cytokine expression.

## 1. Introduction

Sepsis remains an important healthcare challenge affecting 300 cases per 100,000 in the United States each year. A fourth of these cases may develop severe sepsis with septic shock, resulting in 50% mortality [1,2]. Between 19 and 48.9 million cases of sepsis per year are estimated to occur worldwide, and in 2017, the World Health Organization recognized sepsis as a global health priority [3]. The lungs are often affected in sepsis, resulting in acute lung injury characterized by inflammation, which leads to respiratory failure, a major cause of morbidity and mortality in humans and rodents [4,5]. Many of the manifestations of sepsis have been ascribed to lipopolysaccharide (LPS), a bacterial toxin found on the outermost membrane of many bacteria, especially gram-negative bacteria [6,7]. This has prompted work towards the development of strategies capable of modulating its effects, including anti-LPS antibodies. Unfortunately, these interventions have not led to improved outcomes in patients with sepsis [8]. The later discovery of toll-like receptor-4 (TLR-4) suggested a novel target [9]. However, despite the perceived importance of these pathways, a trial of a TLR-4 antagonist, eritoran, failed to improve patients [10]. Thus, to date, efforts to improve sepsis outcomes by targeting LPS- or TLR-4- related pathways have not yielded effective interventions.

One possible way that LPS could promote lung inflammation is through the induction of redox stress. LPS-challenged mice have been shown to undergo oxidation of their plasma redox state (Eh) for the thiol disulfide couple cysteine (Cys) and cystine (CySS), expressed as Eh Cys/CySS [11,12]. Eh Cys/CySS is operative extracellularly, as the Cys/CySS thiol disulfide redox couple is the predominant low-molecular-weight thiol disulfide pool found in plasma, and it controls intracellular levels of glutathione [13,14]. Changes in the redox potential of extracellular thiol pairs serve as independent transducers of oxidant stress. The novelty of this signaling mechanism is that it can trigger intracellular redox signaling by oxidation of membrane-bound proteins [15]. Redox stress via Eh Cys/CySS oxidation has been shown to activate the p44/p42 MAPK pathway in Caco-2 cells, which are associated with increased cell proliferation [16]. In endothelial cells, an oxidized Eh Cys/CySS is sufficient to activate cellular reactive oxygen species (ROS) generation [17]. Lung fibroblasts cultured in media with oxidized Eh Cys/CySS undergo myofibroblast transdifferentiation and are stimulated to produce TGFβ, fibronectin, and collagen [18]. Considering these observations, we propose that Eh Cys/CySS oxidation is a major driver of LPS-induced lung injury in sepsis. Yet, how exactly redox stress related to Eh Cys/CySS oxidation is sensed by immune cells remains unclear.

We previously reported that α4 nicotinic acetylcholine receptors (nAChRs) serve as sensors of redox stress in lung fibroblasts [19]. Specifically, we observed that intracellular signaling triggered by Eh Cys/CySS oxidation was inhibited by pharmacological or genetic inhibition of α4 nAChR expression. The importance of these receptors in tissue injury was recently highlighted by our work showing that α4 nAChR knockout animals are protected against alcohol-induced liver injury [20], but their role in lung injury remains untested.

α4 nAChRs are members of the nAChR family of multimeric acetylcholine-triggered cation channel proteins expressed in neuronal and non-neuronal tissues [21]. At least sixteen genes that code for nAChRs have been identified to date, including four β subunits (β1–β4) and nine distinct α subunits (α1–α7, α9, and α10). In the lungs, nAChRs are expressed in epithelial cells, fibroblasts, and immune cells, and have been implicated in regulation of lung development, lung tissue remodeling, and lung cancer [22,23,24,25,26,27,28,29,30]. Like other nAChRs, α4 nAChRs organize into pentameric proteins at the cell surface, but while α7 nAChRs, for example, are homopentamers made up of five α7 subunits, α4 nAChR sub-units are found to be associated with other nAChR subunits such as β2 [31]. Thus, these receptors form heteropentamers, but the partner(s) of α4 subunits in the lungs has(ve) not been defined. For simplicity, we will refer to such α4-containing nAChRs as α4 nAChRs, while recognizing the more complex nature of these receptors.

Here, we test the hypothesis that α4 nAChR signaling in monocytic cells is activated by LPS-induced redox stress, which results in the upregulation of proinflammatory cytokines that drive lung inflammation in the setting of endotoxemia.

## 2. Results

### 2.1. LPS Activation of Human THP-1 Monocytic Cells Is Dependent on α4 nAChRs

**Figure 1:**
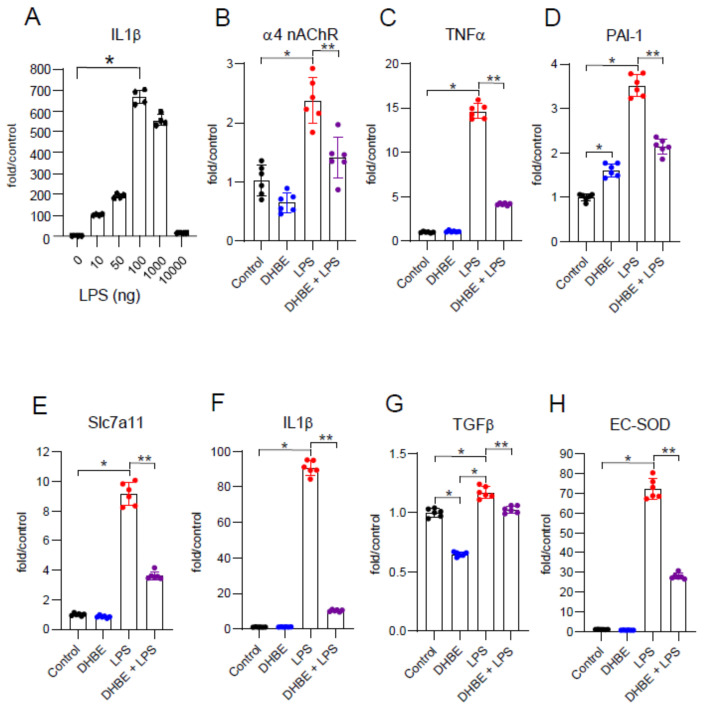
Effects of LPS in THP-1 cells. (**A**) Human monocytic cells, THP-1, were initially treated with varying doses of LPS (0–10,000 ng, 24 h) and found to have optimal response at 100 ng. Thus, further experiments were performed with that dose. (**B**–**H**) Treatment with LPS (100 ng/mL, 24 h) showed increased mRNA expression of α4 nAChRs and several oxidative stress and inflammatory genes including TNFα, PAI-1, Slc7a11, IL-1β, TGFβ, and EC-SOD compared with non-LPS-treated control cells. Results of 2-Way ANOVA followed by post hoc analysis of comparisons between groups are shown (Tukey’s multiple comparison test). Results expressed as the fold increase in mRNA compared with samples from non-treated control cells using the 2^−ΔΔCt^ method. * *p* < 0.0002 when compared with non-treated control cells. ** *p* < 0.0002 when compared with LPS-treated cells.

To begin to determine whether α4 nAChRs were involved in mediating responses to LPS in macrophages, we employed an in vitro model. Human THP-1 monocytic cells were treated with increasing concentration of LPS (0–1000 ng/mL) for 24 h. At this time point, the highest expression of IL-1β was seen with 100 ng/mL; subsequent experiments were performed with this concentration (Figure 1A). Exposure to LPS resulted in an increase in α4 nAChR mRNA levels, suggesting the upregulation of these receptors in the setting of LPS stimulation (Figure 1B). This effect of LPS was blocked by the addition of an α4 nAChR-selective antagonist, DHβE. We also observed that LPS stimulated the mRNA expression of relevant genes, including TNFα, plasminogen activator inhibitor-1 (PAI-1), the cysteine co-transporter solute carrier family 7 member 11 (Slc7a11), IL-1β, the profibrotic growth factor transforming growth factor-β1 (TGFβ1), and extracellular superoxide dismutase (EC-SOD), (Figure 1C–H). Consistent with our hypothesis, DHβE inhibited or ameliorated the LPS-induced expression of these genes, suggesting a role for α4 nAChRs in LPS-related events.

### 2.2. Effects of LPS In Vivo

**Figure 2:**
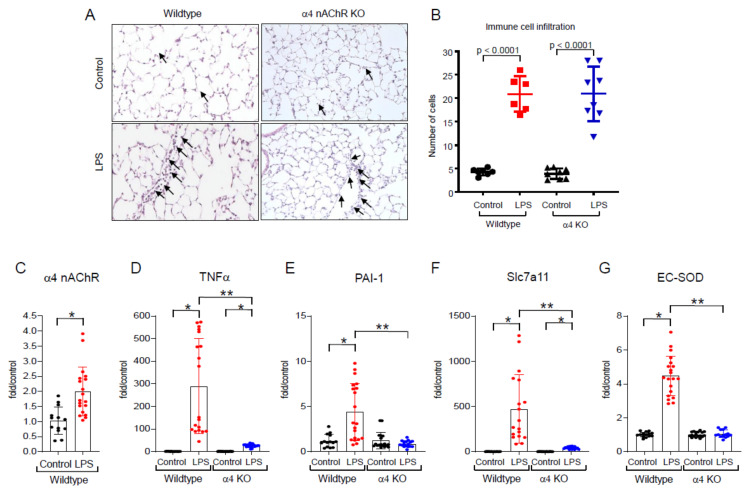
Effects of LPS in the lungs of α4 nAChR KO mice. (**A**) Lung tissue from LPS-treated animals (3 μg/g IT, 24 h) showed increased inflammation as demonstrated by an influx in monocytic cells (20× magnification). Some cells are depicted with arrows. (**B**) The findings did not differ between WT and knockout animals. (**C**–**G**) Lung tissue isolated from LPS (10 mg/kg, 4 h)-treated wild-type animals showed increased expression of α4 nAChR, TNFα, PAI-1, Slc7a11, and EC-SOD mRNA when compared with control non-treated mice. The α4 nAChR KO mice treated with LPS demonstrated a significant reduction in the expression of TNFα, PAI-1, EC-SOD, and Scl7a11 when compared with wild-type animals. Data were analyzed as described for Figure 1. * *p* < 0.0002 when compared with non-treated control cells. ** *p* < 0.0002 when compared with LPS-treated cells.

To further test the hypothesis in vivo, we generated animals with global knockout mutations of α4 in the C57BL/6 background using CRISPR/Cas9 technology. Mice homozygous for a 17 bp deletion in the α4 nAChR exon 4 were generated for study; these animals have been characterized and appear to have a normal phenotype at baseline [20]. As presented in Figure 2A,B), the administration of LPS in WT animals induced mild influx of inflammatory cells into the lungs (some cells, not all, are highlighted with arrows). Interestingly, the number of immune cells identified in the lungs of α4 nAChR-deficient animals did not differ from the cells counted in the lungs of LPS-treated WT animals (Figure 2B). Nevertheless, the lungs of LPS-treated WT animals were obtained and found to show increased expression of α4 nAChRs (Figure 2C). As with our observations in THP-1 cells, LPS also induced the expression in the lungs of mRNAs coding for TNFα, PAI-1, Slc7a11, and EC-SOD in WT animals. These genes were not elevated in the lungs harvested from α4 nAChR knockout animals (Figure 2D–G).

### 2.3. LPS-Induced Redox Stress Results in α4 nAChR Activation and Signaling

**Figure 3:**
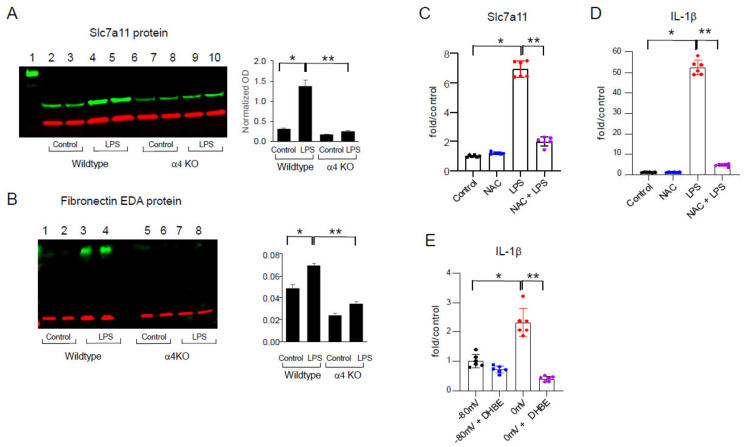
THP-1 cells after LPS stimulation and exposure to redox stress. (**A**,**B**) Lung tissue isolated from LPS-treated wild-type animals showed increased expression of Slc7a11 and fibronectin EDA protein (green color; red color for control gene) when compared with untreated animals, while α4 nAChR knockout animals failed to show this effect. (**C**) THP-1 cells treated with LPS (100 ng/mL, 24 h) showed induction of Slc7a11 mRNA. This effect was inhibited by N-acetylcysteine (NAC, 5 mM). * *p* < 0.0001 when compared with non-treated control cells. ** *p* < 0.0001 when compared with LPS. (**D**) THP-1 cells treated with LPS showed induction of IL-1β mRNA expression. This effect was inhibited by N-acetylcysteine (NAC, 5 mM). * *p* < 0.0001 when compared with non-treated control cells. ** *p* < 0.0001 when compared with LPS. (**E**) Redox stress (Eh Cys/CySS 0 mV)-induced IL-1β, which was inhibited by the α4 nAChR inhibitor, DHβE (100 μM). * *p* < 0.0001 when compared with −80 mV media. ** *p* < 0.0001 when compared with 0 mV media.

The above studies suggested that LPS acts through α4 nAChRs both in vitro and in vivo. Previously, it was demonstrated that LPS administration in rodents led to redox stress resulting from oxidation of the Eh Cys/CySS, which induced cytokine expression [11,12]. Thus, we hypothesized that redox stress generated by LPS is responsible for the activation of α4 nAChRs, which ultimately triggers proinflammatory gene expression. Consistent with this idea was the demonstration that LPS stimulated the mRNA (Figure 2F) and protein expression (Figure 3A) of Slc7a11, a component of the cystine-glutamate antiporter, in the lungs of WT animals, but this effect was inhibited in α4 nAChR knockout animals. The same observation was made when examining the matrix glycoprotein fibronectin EDA, also known to be stimulated in LPS-induced lung inflammation (Figure 3B) [32].

To investigate this further, we tested THP-1 cells and again demonstrated induction of Slc7a11 mRNA in LPS-treated cells (Figure 3C). Importantly, this effect was inhibited by the thiol antioxidant N-acetylcysteine, emphasizing the role of oxidative stress in this model. Similar results were shown for IL-1β mRNA expression (Figure 3D). We then exposed the cells to media with normal (−80 mV) versus oxidized Eh Cys/CySS (−0 mV) and showed that this treatment mimicked the effects of LPS by inducing IL-1β mRNA expression. As noted with LPS, redox stress induction of IL-1β was also inhibited by the α4 nAChR inhibitor DHβE (Figure 3E).

**Figure 4:**
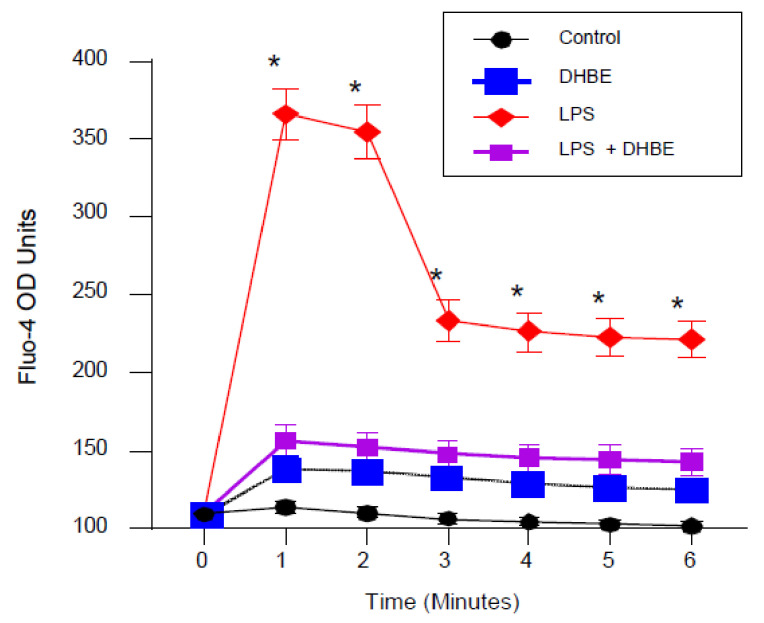
LPS induced calcium influx in THP-1 cells. THP-1 cells were treated with LPS (100 ng/mL, 0–6 min) followed by calcium influx detection. LPS induced calcium influx within 1 min after exposure while DHβE alone had no effect. However, DHβE (100 μM) inhibited the induction of calcium influx in cells exposed to LPS. * *p* < 0.0002 when compared with non-treated control, DHβE, and LPS + DHβE cells.

The above observations suggested that LPS-induced redox stress triggers cytokine expression in an α4 nAChR-dependent manner, but exactly how α4 nAChR activation stimulates cytokine exposure remains unexplored. NAChRs are multimeric cation channel proteins known to gate the entry of calcium into cells [33]. Thus, we tested the effects of LPS on calcium flux in THP-1 cells. As presented in Figure 4, we found that LPS stimulated calcium influx in THP-1 cells. Notably, this effect was inhibited by the α4 nAChR inhibitor DHβE.

## 3. Discussion

Our studies explored the hypothesis that LPS-related redox stress induces lung inflammation by activating α4 nAChR signaling. Consistent with this hypothesis, we found that LPS induced the expression in human THP-1 monocytic cells of the proinflammatory cytokines TNF-α and IL-1β, as well as the expression of the profibrotic growth factor TGFβ1, PAI-1, EC-SOD, and the matrix glycoprotein fibronectin EDA. These effects were blocked by pharmacological inhibition of α4 nAChRs with DHβE. The potential role of α4 nAChRs was further highlighted by the observation that LPS induced the expression of this receptor, a process also inhibited by DHβE. We then demonstrated the induction of relevant molecules in the lungs of LPS-treated wild-type animals, but not in animals with a global knockout mutation in the α4 nAChR. Notably, although α4 nAChR expression affected immune cell activation, it did not appear necessary for the influx of immune cells into the lungs of LPS-treated animals. Emphasizing the importance of redox stress in these processes, we showed that LPS-induced IL-1β expression was inhibited by N-acetylcysteine and was mimicked by Eh Cys/CySS oxidation, and the latter was also inhibited by DHβE. Finally, we showed that LPS induced calcium influx in THP-1 cells in an α4 nAChR-dependent manner.

To our knowledge, this is the first investigation into the potential role of α4 nAChRs in LPS-related lung inflammation. Several functional nAChR units are expressed in the lungs, including α3, α5, and α7 nAChR, which have been detected in both human and mouse bronchial epithelial cells [22]. These and related receptors have been implicated in lung physiology and disease [24,25,26]. NAChRs have been detected in alveolar epithelial cells, and in immune cells and lung fibroblasts ([22,23,29,30,34], this report), but their role in physiological pulmonary processes is less well understood. Yet, an important role in tissue injury is likely, as demonstrated by our own work showing that α4 nAChRs mediate alcohol-induced liver injury [20].

**Figure 5:**
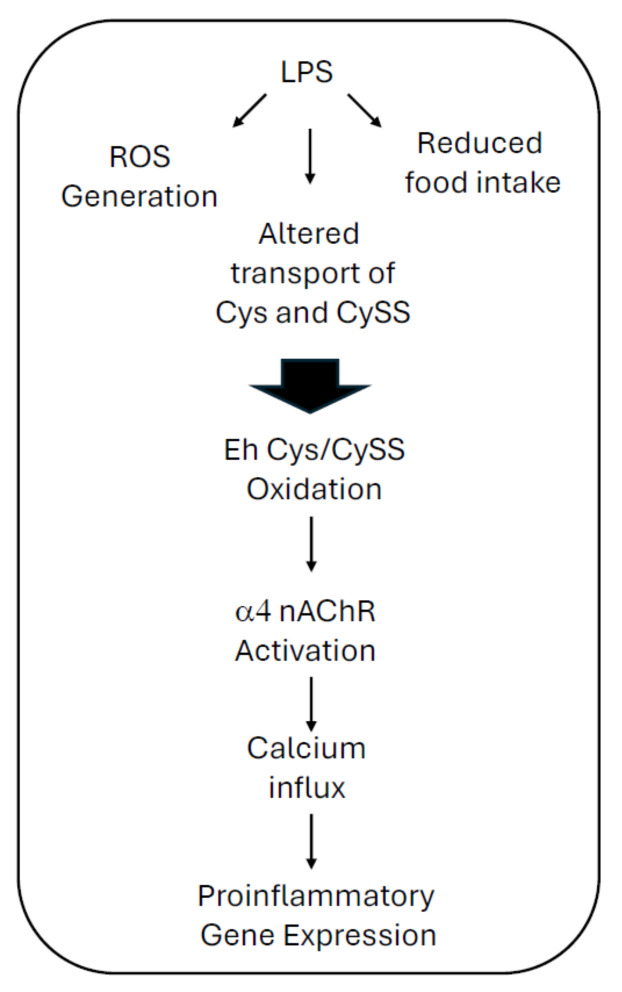
LPS-induced redox stress and α4 nAChR-dependent lung inflammation. It has been postulated that LPS induces oxidation of the plasma Cys/CySS redox state in animals by a combination of three mechanisms: early generation of reactive oxygen species, altered transport of Cys and CySS, and reduced food intake [11,12]. The resulting oxidized Eh Cys/CySS triggers α4 nAChR activation and signaling through calcium influx. The latter results in the expression of proinflammatory genes.

Our studies highlight the potential role of cholinergic signaling in immune cells in lung inflammatory processes. Immune cells express many if not most of the components of the cholinergic system, including acetylcholine, choline acetyltransferase, acetylcholinesterase, and both muscarinic and nicotinic acetylcholine receptors [35]. However, little is known about how nicotinic acetylcholine receptors influence pulmonary inflammation. Another intriguing concept presented in the current manuscript is that α4 nAChR signaling is triggered by LPS-induced redox stress. Exactly how LPS induces Eh Cys/CySS oxidation is not clear. In rats, Iyer and colleagues found a decrease in plasma Cys and an increase in CySS just two hours after LPS administration (before noticeable lung inflammation) [12]. The total plasma pool of Cys and CySS did not change, suggesting amino acid modifications were not affected by their transport early on. Six hours later, however, they observed a significant decrease in CySS, while the overall Eh Cys/CySS remained oxidized for the next 48 h, resulting in a 50-mV difference. Consistent with our observations, they found that this latter change was related to induction of Slc7a11, a component of the xCT- co-transporter that imports CySS into cells [12]. These findings are intriguing, considering that we previously found that decreased expression of Slc7a11 in cultured fibroblasts triggers redox stress [36]. This highlights the complexity of redox reactions in lungs and that more work is needed to define how this system influences distinct cells in healthy and diseased lungs. Nevertheless, based on their findings, the authors concluded that Eh Cys/CySS oxidation early after LPS administration precedes pathologic changes in lungs and occurs without changes in the total pool size of Cys and CySS. In contrast, Eh Cys/CySS oxidation after six hours occurred with a decrease in total pool size, suggesting that sustained oxidation was a consequence of decreased precursor availability relative to tissue demand [12]. They also postulated that decreased food intake influenced these latter events, although food intake was not evaluated in that study. These observations are important, as sustained LPS-induced redox stress and poor food intake may perpetuate the deleterious events observed in endotoxin-related sepsis [12] (Figure 5).

In our experiments, N-acetylcysteine inhibited the effects of LPS on THP-1 cells, perhaps by affecting the redox potential of thiols or indirectly by increasing levels of glutathione and scavenging of reactive species. Others have demonstrated the ability of NAC to attenuate LPS-induced responses in bronchial epithelial cells [37]. Specifically, they found that LPS-induced intracellular calcium influx leads to NADPH oxidase activation and generation of intracellular ROS production, which was attenuated by N-acetylcysteine. Notably, these events were not inhibited by a TLR4 inhibitor. Instead, they were mediated through transient receptor potential ankyrin 1 (TRPA1), a Ca^2+^ permeant channel [37]. This emphasizes the idea that LPS can induce effects via mechanisms other than TLR4 activation. We have not determined the relationship between TRPA1 and α4 nAChRs in our system.

Considering the perceived importance of the above observations, we focused our attention on how redox stress triggers lung inflammation. We first considered the possibility that α4 nAChRs were involved in these processes based on earlier studies designed to test the effects of alcohol in lung fibroblasts [19]. This model is relevant, since chronic alcohol exposure promotes acute lung injury in at-risk settings such as sepsis [38,39]. We observed that alcohol activated fibroblasts through ligand-independent mechanisms and this effect was inhibited by antioxidants and mimicked by media with oxidized Eh Cys/CySS [19]. These earlier studies suggested that α4 nAChRs might serve as ‘sensors’ of redox stress. The exact mechanisms by which redox stress activates α4 nAChRs remain unclear, but we speculate that conformational changes in α4 subunits driven by modifications in cysteine residues strategically located near the extracellular ligand-binding domain of α4 subunits prompt this change [40]. In turn, the opening of the membrane channel allows the influx of cations. This concept remains speculative and requires further investigation.

Independent of the mechanisms by which α4 nAChRs are activated by redox stress, their signaling leads to downstream events responsible for differential gene expression. Here, we demonstrated upregulation of α4 nAChRs, suggesting the ability of α4 nAChR signaling to stimulate its own expression. We also showed the induction of the proinflammatory cytokines TNFα and IL-1β, perhaps through upregulation of NFκB, but this needs further testing [41,42]. In addition, we observed the upregulation of the protease inhibitor PAI-1, the antioxidant EC-SOD, and the profibrotic growth factor TGFβ1. These molecules were tested because they have been implicated in the pathophysiology of LPS-induced tissue injury [34,43,44,45,46]. We also found upregulation of Slc7a11, which, as previously mentioned, has been implicated in regulation of redox stress after LPS exposure [12]. Slc7a11 is a subunit of the system Xc^-^ glutamate-CySS antiporter and animals deficient in this molecule show Eh Cys/CySS oxidation [47]. In prior work, we showed that lung fibroblasts deficient in Slc7a11 are unable to compensate for redox stress, a deficit that improves with genetic overexpression of the gene [36,48]. Interestingly, decreased expression of Slc7a11 in lung fibroblasts triggers a profibrotic and senescence phenotype, which is reversible via upregulation of Slc7a11 via pharmacologic and genetic means [49]. We speculate that in our system, upregulation of Slc7a11 in cells stimulated with LPS might represent a compensatory mechanism triggered to counter the early redox stress generated in the setting of endotoxemia, or perhaps upregulation of Slc7a11 promotes disruption of other critical pathways, thereby leading to tissue injury, but this requires further investigation.

One intriguing observation was that α4 nAChR expression was not necessary for the influx of immune cells into the lungs of LPS-treated animals, as a similar number of immune cells were counted in the lungs of LPS-treated WT and knockout animals (Figure 2). This suggests that α4 nAChRs may not be involved in mediating a number of processes related to LPS-induced immune cell recruitment such as endothelial cell activation, cell-cell and cell-matrix interactions, and the release of chemoattractants by resident cells in lungs. These observations deserve further investigation, but our data to date suggest that mechanisms of immune cell recruitment and immune cell cytokine expression of newly recruited immune cells are distinct from one another.

Our study has limitations in that it does not test the possibility that other nAChRs compensate for the lack of α4 nAChRs. For example, α7 nAChR activation results in the deployment of anti-inflammatory signals and its stimulation has been shown to inhibit LPS-induced TNF-α expression in murine lungs [50]. In contrast, others have found that α7 nAChRs may promote inflammation and extracellular matrix remodeling in other settings [51,52]. Thus, α7 and α4 nAChRs may play contrasting roles in LPS-induced lung injury and other inflammatory processes. It is also conceivable that α4 nAChRs play distinct roles depending on the source of the lung injury. For instance, Kiguchi et al. reported that activation of α4β2 nAChRs with the agonist TC-2559 inhibits pSTAT3 in vitro, thereby inhibiting LPS-TLR4 induction of pp65 and IL1β production [53]. These observations contrast with ours, but differences in experimental design may explain these observations. Importantly, our data generated both in vitro and in vivo suggest that α4 nAChRs indeed mediate the detrimental effects of LPS.

In conclusion, our studies suggest that LPS might promote lung injury by inducing redox stress. In turn, redox stress activates α4 nAChR signaling, leading to the upregulation of proinflammatory and tissue remodeling pathways. The availability of new agents against nAChRs (e.g., varenicline) suggests that these receptors could be targeted in the future by therapeutic interventions at the clinic, but further preclinical studies in relevant models are needed to fully define their potential [54]. Other interventions may target redox stress, as has been observed for diets containing distinct concentrations of sulfur amino acids capable of reducing Eh Cys/CySS [55]. Further studies will be needed to evaluate the significance of these findings in humans.

## 4. Materials and Methods

### 4.1. Reagents

All reagents were purchased from Sigma Chemicals (St. Louis, MO, USA) or Fisher Scientific (Pittsburgh, PA, USA) unless otherwise specified. Dihydro-β-erythroidine hydrobromide (DHβE) was purchased from Bio-techne (Minneapolis, MN, USA). DHβE is a potent competitive antagonist of nAChRs with specific selectivity for α4β4 and α4β2 nAChRs.

### 4.2. Cell Culture

Human monocytic cells, THP-1 (ATCC TIB-202, Rockville, MD, USA), were maintained in RPMI 1640 medium (Corning, Corning, NY, USA) supplemented with 10% heat-inactivated fetal bovine serum (FBS) and 1% antibiotic-antimycotic solution (100 U/mL penicillin G sodium, 100 U/mL streptomycin sulfate, 0.25 mg/mL amphotericin B) and incubated in a humidified 5% CO_2_ incubator at 37 °C.

### 4.3. Generation of α4 nAChR Knockout Mice

The α4 nAChR knockout animals were generated at the Transgenic Mouse Facility, University of California, using CRISPR/Cas9 as previously described [20]. The α4 nAChR knockout animals were then backcrossed with wild-type (WT) C57BL/6J mice (Jackson Laboratories, Bar Harbor, ME, USA) for a minimum of 8 generations. All animals were housed in a pathogen-free facility with a 12 h light/dark cycle and access to food and water ad libitum. The institutional animal care and use committee at Thomas Jefferson University approved all animal experiments.

### 4.4. LPS-Induced Lung Injury

LPS (10 mg/kg *E. coli* 0111:B6, IP) was administered to wild-type and α4 nAChR knockout (α4KO) mice, followed by harvesting the lungs for analysis of markers of inflammation and oxidative stress. The animals were injected with endotoxin, which has been shown to cause lung inflammation, edema, and fibronectin expression peaking at 6–24 h [11]. The animals were euthanized at 4 h. After inflation and perfusion of the left lobes with fixative, the lungs were harvested and processed for further study.

### 4.5. Isolation and Measurement of mRNA by qPCR

Lung tissue was harvested and placed into sterile DNase- and RNase-free tubes containing 1.4 mm ceramic beads (Omni International, Kennesaw, GA, USA) and 700 µL Ribozol RNA isolation reagent (Amresco, Solon, OH, USA). Samples were homogenized for 30 s using a Bead Mill 4 (Fisher Scientific, Pittsburgh, PA, USA) and centrifuged (10,000× *g*) for 5 min. RNA was isolated from the supernatant and purified using Zymo Direct-zol RNA mini-prep plus (Zymo Research, Irvine, CA, USA). The purified RNA was quantified using a Beckman Coulter DU800 spectrophotometer. RNA was reverse-transcribed using the Bio-Rad iScript cDNA synthesis kit (Bio-Rad, Hercules, CA, USA). Real-time quantitative PCR (qPCR) was performed for the quantification of Slc7a11, Tnf, Serpine1 (PAI-1), Chrna4, EC-SOD, TGFβ, IL1β, and 18S ribosomal mRNA expression using TaqMan Gene Expression Assay (human Slc7a11, Hs00921938_m1; Tnf, Hs00174128_m1; Serpine1, Hs00167155_m1; Chrna4, Hs00181247_m1; Sod3, Hs00162090_m1; Tgfβ, Hs00998133_m1; IL1β, Hs01555410_m1; 18S, Hs03003631_g1; or mouse Slc7a11, Mm00442530_m1; Tnf, Mm00443258_m1; Serpine1, Mm00435858_m1; Chrna4, Mm00516561; EC-SOD, Mm01213380_s1; IL1β, Mm00434228_m1; 18S, Mm03928990_g1 (Life Technologies, Carlsbad, CA, USA)) according to the manufacturer’s instructions. The Applied Biosystems QuantStudio3 Real-Time PCR System (Waltham, MA, USA) was used with the following reaction settings: 50 °C for 2 min, 95 °C for 0 min, followed by 40 cycles of 95 °C for 15 s and 60 °C for 1 min. Results were analyzed using QuantStudio Analysis Software version 1.3 and the amplification curves were subjected to the mathematical equation of the second derivative. Ct values were normalized to the housekeeping gene 18S. Results were expressed as the fold increase in mRNA compared with samples from control-treated THP-1 cells or wild-type pair-fed mice using the 2^−ΔΔCt^ method.

### 4.6. Western Blot

Lung tissue was harvested in RIPA buffer (Thermo Fisher Scientific, Waltham, MA, USA) and homogenized using a Bead Mill 4 cell disruptor (Thermo Fisher Scientific, Waltham, MA, USA) and 1.4 mm ceramic beads (Omni International, Kennesaw, GA, USA). Total protein concentration was determined using the Bradford protein dye reagent (Bio-Rad, Hercules, CA, USA) and a Beckman DU800 spectrophotometer (Beckman Coulter, Brea, CA, USA) at OD 595. Protein samples (20 mg) were loaded onto a 4–20% Mini-Protean TGX Precast Gel and electrophoresed at 150 V for 1–3 h. Afterward, protein was transferred onto 0.2 mm nitrocellulose membrane (Bio-Rad, Hercules, CA, USA) at 25 V for 1.5 h using a Transblot SD semidry transfer cell (Bio-Rad, Hercules, CA, USA). Membranes were blocked for 1 h at room temperature in Odyssey Blocking Buffer (LI-COR Biosciences, Lincoln, NE, USA) and incubated overnight at 4 °C with primary antibody against GAPDH (Abcam, No. ab8245), Slc7a11 (Abcam, No. 175186, 1:1000 dilution), or FN EDA (Sigma, No. SAB4200784, 1:1000 dilution). Membranes were washed (3 × 10 min) with PBS containing 0.2% Tween-20 (PBST) and then incubated with secondary antibody (LI-COR, goat anti-rabbit, No. 926-32211 or goat anti-mouse, No. 926-68070 at 1:5000 or 1:20,000 dilution, respectively) for 1 h at room temperature. Membranes were washed in PBST (3 × 10 min) and scanned using a LI-COR Odyssey CLx imaging system and analyzed in Image Studio Lite (LI-COR Biotechnology, Lincoln, NB, USA).

### 4.7. Histology and Myeloperoxidase Staining

The mouse lungs were fixed in buffered formalin (Fisher Scientific, Pittsburgh, PA, USA) overnight at room temperature, dehydrated and embedded in paraffin. Tissue sections (6 μM) were deparaffinized and antigen retrieval was performed using Trilogy reagent (Fisher Scientific, Pittsburgh, PA, USA). Sections were stained with an antibody to myeloperoxidase (MPO; ab208670, Abcam, Waltham, MA, USA) at a dilution of 1:500. Images were taken using an EVOS FL microscope (Life Technologies, Carlsbad, CA, USA) at 20× magnification. Three mice from each group were analyzed with 4 fields randomly selected from each tissue section, and cells positive for MPO were counted. The average number of positive cells per field for each mouse were used to compare groups using statistics in the GraphPad Prism program version 9.3.1.

### 4.8. Calcium Influx Assay

THP-1 cells were harvested by centrifugation at 200× *g* for 3 min. The medium was removed, and the cells resuspended in Fluo-4 assay buffer (Fluo-4 NW Calcium Assay Kit, Molecular Probes, Eugene, OR, USA) at a density of 2.5 × 10^6^ cells/mL in a total volume of 50 µL per well of a 96-well cell culture plate. The same volume of the assay buffer alone was used for no-cell control wells. The plates and cells were incubated at 37 °C and 5% CO_2_ for 60 min prior to the addition of 50 µL of 2x dye-loading solution. The cells were incubated at 37 °C for 30 min, then at room temperature for an additional 30 min. The cells were finally treated with LPS (100 ng/mL) in the presence or absence of the α4 nAChR antagonist, dihydro-β-erythroidine hydrobromide (DHβE, 100 μM). The fluorescent calcium influx was measured using a BioTek Synergy HT Multi-Mode Microplate Reader (Agilent, Santa Clara, CA, USA) at 494 nm. Each treatment included ten separate technical replicates. Results were expressed as relative Fluo-4 optical density units measured at 494 nm.

### 4.9. Analysis of Data and Statistical Evaluation

All assays were performed on at least 4, and up to 24, biological replicates for each sample group. Numerical data are presented as means plus standard error of the mean. Significance was assessed by 2-way ANOVA using Tukey’s multiple comparison test in GraphPad Prism version 9.3.1 to compare individual groups. For graphical representation of significance between groups, the following notations were used: *p* < 0.0332 (*), *p* < 0.0021 (**), *p* < 0.0002 (***), and *p* < 0.0001 (****).

## Data Availability

Data available upon formal request to J Roman.
